# Transforming healthcare using big data: a systematic and bibliometric review

**DOI:** 10.3389/fdgth.2026.1807970

**Published:** 2026-07-02

**Authors:** Javier Gamboa-Cruzado, Kiara Fernandez-Perez, Herber Laura-Abarca, Cristina Alzamora Rivero, Rodolfo Cubas Agreda, Alex Salazar-Marzal, Amanda Durán Carhuamaca, Ángel Nuñez Meza

**Affiliations:** 1 Facultad de Ingeniería Industrial y de Sistemas, Universidad Nacional Federico Villarreal, Lima, Peru; 2 Facultad de Ciencias Administrativas, Universidad Nacional Mayor de San Marcos, Lima, Peru; 3 Facultad de Ingeniería, Universidad Nacional de Cañete, Cañete, Peru; 4 Facultad de Ingeniería de Sistemas, Universidad Nacional Daniel Alcides Carrión, Pasco, Peru

**Keywords:** complex data, health system, healthcare, massive datasets, systematic and bibliometric review

## Abstract

**Introduction:**

The application of Big Data in healthcare enhances disease management by improving diagnostics and treatments, offering a foundation for positive impacts on the health of diverse populations.

**Objective:**

This paper aims to analyze the impact of Big Data on healthcare through a comprehensive review of scientific papers that explore its application in the management, diagnosis, and treatment of various diseases, assessing its benefits, challenges, and potential to revolutionize healthcare systems.

**Methods:**

The study compiled 63 papers from sources such as Web of Science, IEEE Xplore, Scopus, ScienceDirect, and ACM Digital Library, conducting an exhaustive systematic review of publications from 2018 to 2024 on the use of Big Data in healthcare.

**Results:**

The findings indicate that prediction and decision-making are the most relevant criteria for evaluating the effectiveness of Big Data in healthcare. Additionally, it was observed that most research is published in journals ranked at the Q1 quartile level.

**Discussion:**

Insights are provided on how Big Data criteria can optimize healthcare delivery, to guide evidence-based decision-making and healthcare system optimization.

## 1 Introduction

Big Data has established itself as a fundamental tool in healthcare, providing advanced capabilities for analyzing large volumes of medical data. In this paper, Big Data is understood as the large-scale, heterogeneous, and rapidly generated set of data produced from multiple healthcare-related sources, including electronic health records, clinical repositories, biomedical devices, wearable sensors, medical imaging systems, administrative databases, and digital health platforms. In healthcare, its value lies not only in the volume of available data, but also in the capacity to integrate, process, and analyze complex information to support clinical decision-making, risk prediction, resource management, disease diagnosis, personalized care, and healthcare system optimization (17, 22, 63). Its implementation enhances diagnostic accuracy and personalizes treatments by identifying patterns and trends in population health. By integrating information from medical records and epidemiological studies, it facilitates outbreak anticipation, optimizes resource allocation, and promotes more effective preventive strategies. These comprehensive analytical capabilities drive more efficient and proactive medical care, positively impacting the well-being of diverse communities. Big Data analytics has demonstrated significant potential to transform health management and services, though it faces considerable challenges regarding privacy, security, and handling large datasets (2, 65, 66). Authors (2, 3, 17, 68) emphasize that, beyond optimizing diagnostics and treatments, Big Data can predict epidemics, improve health sustainability, and foster more informed decision-making in both administrative and clinical settings.

Additionally, the use of wearable sensors and MEMS systems has generated significant amounts of data, which, combined with artificial intelligence, enable more precise clinical decisions. In this context, recent studies propose a five-layer IoHT framework to optimize resources and ensure high-quality service in e-health systems (4, 10, 67). Advances in deep learning and Radiomics have also improved diagnostic accuracy, particularly through convolutional neural networks for automated biomarker extraction (11, 76).

Furthermore, the integration of Big Data Analytics (BDA) in healthcare organizations has been proposed to optimize both administrative and clinical decisions, highlighting its ability to improve diagnostic and treatment quality through the analysis of structured and unstructured data (17, 19, 20). Authors (14, 15, 19) underscore the relationship between BDA capabilities and the quality of health services, while others (9, 18) present frameworks like DeepMist, which combines fog computing and deep learning to predict diseases with high accuracy.

Recent research has also focused on digital transformation and its impact on risk management, sustainability, and resource planning in healthcare, emphasizing the use of Big Data to incorporate environmental factors into decision-making processes (19, 70, 71). Telemedicine has proven to be a key tool in addressing barriers to healthcare access in rural areas during the COVID-19 pandemic, ensuring continuity of health services and improving disease management through digital technologies and innovative approaches (68). In the healthcare context, digital transformation has also been associated with the expansion of telemedicine, mobile health solutions, and data-driven decision-making processes, particularly in emerging economies. However, the adoption of these technologies remains uneven, with persistent gaps in interoperability, digital infrastructure, data governance, and the integration of advanced technologies into routine healthcare services (75). Therefore, this review focuses specifically on Big Data applications that contribute to healthcare management, clinical analytics, digital health infrastructures, and health-system optimization. Finally, bibliometric studies on Big Data in healthcare indicate significant growth in research since 2012, with a notable increase between 2019 and 2021. This analysis identifies key trends and challenges related to global collaboration, standardization, and the implementation of security measures (69, 73, 77).

The literature review reveals that despite advancements in Big Data applied to healthcare, there remains limited exploration of its specific impact on healthcare management. Current studies do not sufficiently delve into the integration of Big Data with administrative and diagnostic processes in hospitals. This paper aims to address these gaps by providing a comprehensive evaluation of how Big Data can optimize management and improve efficiency in the healthcare sector. Consequently, this paper focuses on analyzing the impact of Big Data on healthcare management and assessing its contribution to improving diagnostics and treatments, based on a thorough review of the available literature.

To achieve this, the paper is structured as a systematic review organized as follows: Section II details the methodology applied, Section III presents and discusses the findings of the review, and finally, Section IV provides the conclusions reached along with recommendations for future research.

## 2 Review method

**Figure 1:**
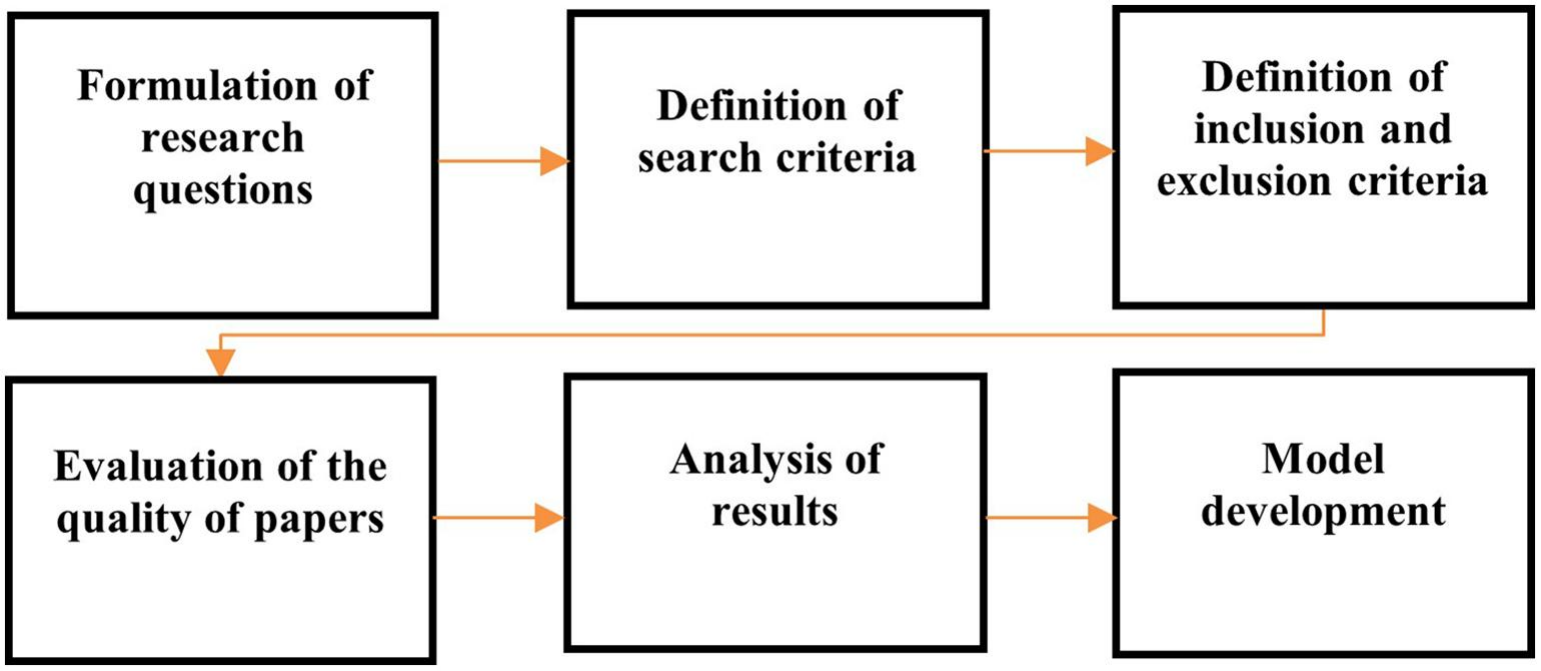
Stages of the SLR.

A Systematic Literature Review (SLR) approach was adopted based on the guidelines of B. Kitchenham (64), as illustrated in Figure 1, with adaptations that include the incorporation of the Preferred Reporting Items for Systematic Reviews and Meta-Analyses (PRISMA) 2020 guidelines (74, 79). This approach enables a comprehensive analysis of the impact of Big Data in healthcare, aiming to address the research questions posed. To ensure the validity of the results, particular attention was given to maintaining a detailed record of the procedure used, which includes creating a database with search equations, exclusion criteria, and the quality assessment employed in the review.

### 2.1 Research main problems and motivation

Given the significance of Big Data research in healthcare, it is essential to implement a search strategy that facilitates the efficient collection and analysis of data from each study. Research questions (RQs) play a fundamental role in this process. To ensure methodological rigor, the formulation of the research questions and the definition of the eligibility criteria were guided by an adapted PICOS framework (Population, Intervention, Comparator, Outcomes, and Study design) tailored to the characteristics of Big Data research in healthcare contexts. Below are five research questions along with their corresponding objectives, summarized in Table 1.

**Table 1:** Research problems and motivation.

Research question	Main motivation
RQ1: What indicators and parameters are used to evaluate the effectiveness of Big Data?	Identify the indicators and parameters used to assess the effectiveness of Big Data
RQ2: What quartile positions do journals occupy that publish research on the impact of Big Data in healthcare?	Investigate the quartile positions of journals publishing research related to the impact of Big Data in healthcare
RQ3: What predominant concepts have been used in research on Big Data and its impact on healthcare?	Categorize the predominant concepts utilized in research on Big Data and its impact on healthcare
RQ4: What key terms tend to co-occur within studies on Big Data and its impact on healthcare?	Analyze the key terms that tend to co-occur in studies on Big Data and its influence on healthcare
RQ5: What main thematic categories are addressed in research on Big Data and its impact on healthcare?	Specify the thematic categories concerning Big Data and its impact on healthcare

### 2.2 Information sources and search strategies

To identify relevant studies, the databases Web of Science, IEEE Xplore, Scopus, ScienceDirect, and ACM Digital Library were utilized. The comprehensive search across all databases was completed on December 18, 2024, thereby establishing the temporal cutoff of the study. Consequently, the 2024 records should be interpreted with caution, as they do not represent a complete publication year and may be affected by indexing delays, shorter exposure time, and limited citation accumulation. The search descriptors were defined as follows:

- Independent variable: “big data”, “complex data”, and “large dataset”.
- Dependent variable: “healthcare”, “health system”, and “medical system”

The search method was carried out using a set of terms designed to facilitate the identification, extraction, and analysis of relevant information. This set, referred to as the search equation, was adapted according to the database used, as specified in Table 2.

**Table 2:** Information sources and search equations.

Source	Search equation
Web of Science	TI = ((“big data” OR “complex data” OR “large dataset”) AND (healthcare OR “health system” OR “medical system”)) OR AB = ((“big data” OR “complex data” OR “large dataset”) AND (healthcare OR “health system” OR “medical system”)) OR AK = ((“big data” OR “complex data” OR “large dataset”) AND (healthcare OR “health system” OR “medical system”))
IEEE Xplore	(((“Document Title”: “big data” OR “Document Title”: “complex data” OR “Document Title”: “large dataset”) AND (“Document Title”: healthcare OR “Document Title”: “health system” OR “Document Title”: “medical system”)) OR ((“Abstract”: “big data” OR “Abstract”: “complex data” OR “Abstract”: “large dataset”) AND (“Abstract”: healthcare OR “Abstract”: “health system” OR “Abstract”: “medical system”)) OR ((“Author Keywords”: “big data” OR “Author Keywords”: “complex data” OR “Author Keywords”: “large dataset”)))
Scopus	TITLE-ABS-KEY ((“big data” OR “complex data” OR “large dataset”) AND (healthcare OR “health system” OR “medical system”))
Science Direct	TITLE-ABSTR-KEY (“big data” OR “complex data” OR “large dataset”) AND (healthcare OR “health system” OR “medical system”)
ACM Digital Library	((Title:“big data” OR Title:“complex data” OR Title:“large dataset”) OR (Abstract:“big data” OR Abstract:“complex data” OR Abstract:“large dataset”) OR (Keywords:“big data” OR Keywords:“complex data” OR Keywords:“large dataset”)) AND ((Title:“healthcare” OR Title:“health system” OR Title:“medical system”) OR (Abstract:“healthcare” OR Abstract:“health system” OR Abstract:“medical system”) OR (Keywords:“healthcare” OR Keywords:“health system” OR Keywords:“medical system”))

### 2.3 Identification and selection of studies

**Figure 2:**
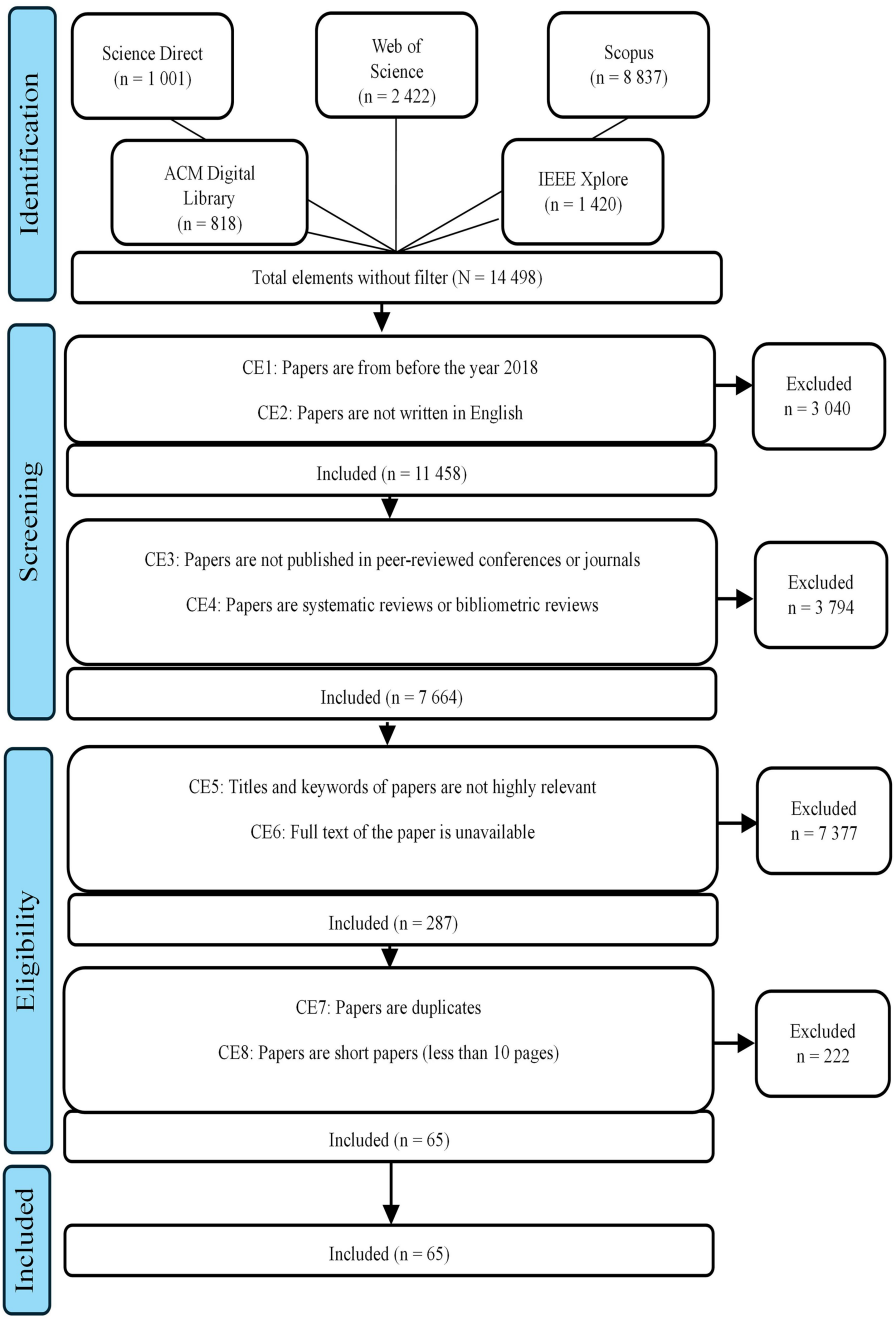
PRISMA flow diagram.

At the conclusion of the search in each information source, a set of relevant studies was compiled, summarized at the top of Figure 2.

To ensure an accurate assessment of the literature's quality, specific exclusion criteria (EC) were defined, enabling the selection of the most relevant papers for the research. These criteria, totaling eight, were applied objectively to obtain an appropriate number of studies. Initially, the keyword-based search identified a total of 14,498 papers. After applying the exclusion criteria, 65 papers were selected.

### 2.4 Quality assessment

In this stage, the selected papers underwent a detailed analysis using seven quality assessment (QA) criteria. During the final selection and filtering process, an official list of included papers was created, ensuring through quality assessment that each study described was both accurate and comprehensible.

QA1: Are the study objectives clearly specified?
QA2: Are the research techniques employed described and justified?
QA3: Is the data collection method adequately detailed?
QA4: Are the collected data adequately explained in the study?
QA5: Is the purpose behind the data analysis clear?
QA6: Are the research questions posed adequately answered?
QA7: Are the links between data, interpretation, and conclusions clearly established?

Each analyzed paper was assessed according to seven quality criteria to evaluate its methodological quality and potential risk of bias. A three-point scale was applied for each criterion: 1 = Poor, when the criterion was insufficiently addressed; 2 = Fair, when the criterion was partially addressed; and 3 = Excellent, when the criterion was clearly and adequately addressed.

To strengthen the consistency of the quality assessment process, the selected papers were evaluated independently by the reviewers. After the individual assessment, the assigned scores were compared, and any discrepancies were discussed until consensus was reached. This procedure was used to reduce subjectivity in the scoring process and to ensure a more transparent and consistent evaluation of the included studies.

The predominance of high scores in Table 3 is explained by the sequential filtering process applied before the quality assessment stage. Only papers that had already met the inclusion criteria, were peer-reviewed, available in full text, written in English, thematically relevant, and aligned with the objectives of the review were subjected to the final quality evaluation. Therefore, the QA stage functioned as a final methodological verification rather than as the initial screening mechanism. Papers scoring below 19 were excluded from the review.

**Table 3:** Quality assessment results.

References	Type	QA1	QA2	QA3	QA4	QA5	QA6	QA7	Score
Abidi et al. (1)	Journal	3	3	3	3	3	3	3	21
Abouelmehdi et al. (2)	Journal	3	3	3	3	3	3	3	21
Ahmad et al. (3)	Journal	3	3	3	3	3	3	3	21
Ahsan et al. (4)	Journal	3	3	3	3	3	3	3	21
Alahmar and Benlamri (5)	Journal	3	3	3	3	3	3	3	21
Alhakami et al. (6)	Journal	3	3	3	3	3	3	3	21
Andry et al. (7)	Journal	3	3	2	3	3	3	3	20
Andry et al. (8)	Journal	3	3	3	3	3	3	3	21
Ar-Reyouchi et al. (9)	Journal	3	3	3	3	3	3	3	21
Asif-Ur-Rahman et al. (10)	Journal	3	3	3	3	3	3	3	21
Badr (11)	Journal	3	3	3	3	3	3	3	21
Bani-Salameh et al. (12)	Journal	3	3	3	3	3	3	3	21
Bansal et al. (13)	Conference	3	3	3	3	3	3	3	21
Basile et al. (14)	Journal	3	3	3	3	3	3	3	21
Basile et al. (15)	Journal	3	3	3	3	3	3	3	21
Batarseh et al. (16)	Journal	3	3	3	3	3	3	3	21
Batko and Ślęzak (17)	Journal	3	3	3	3	3	3	3	21
Bebortta et al. (18)	Journal	3	3	3	3	3	3	3	21
Benzidia et al. (19)	Journal	3	3	3	3	3	3	3	21
Dash et al. (20)	Journal	3	3	3	3	3	3	3	21
El-Sappagh et al. (21)	Journal	3	3	3	3	3	3	3	21
Furstenau et al. (22)	Journal	3	3	3	3	3	3	3	21
Ghaleb et al. (23)	Journal	3	3	3	3	3	3	3	21
Ghaleb et al. (24)	Journal	3	3	3	3	3	3	3	21
Goldstein et al. (25)	Journal	3	3	3	3	3	3	3	21
Hadi et al. (26)	Journal	3	3	3	3	3	3	3	21
Hassan et al. (27)	Journal	3	3	3	3	3	3	3	21
Hsu et al. (28)	Journal	3	3	3	3	3	3	3	21
Hussain et al. (29)	Journal	3	3	3	3	3	3	2	20
Kaur et al. (30)	Journal	3	3	3	3	3	3	3	21
Kazançoğlu et al. (31)	Journal	3	3	3	3	3	3	3	21
Khan et al. (32)	Journal	3	3	3	3	3	3	3	21
Kholaif and Xiao (33)	Journal	3	3	3	3	3	3	3	21
Kim and Chung (34)	Journal	3	3	3	3	3	3	3	21
Krishankumar et al. (35)	Journal	3	3	3	3	3	3	3	21
Lemmen et al. (36)	Journal	3	3	2	3	3	3	3	20
Mary Arockiam et al. (37)	Journal	3	3	3	3	3	3	3	21
Mohapatra et al. (38)	Journal	3	3	3	3	3	3	3	21
Moro Visconti and Morea (39)	Journal	3	3	3	3	3	3	3	21
Navaz et al. (40)	Journal	3	3	3	3	3	3	3	21
Oğur et al. (41)	Journal	3	3	3	3	3	3	3	21
Panayides et al. (42)	Journal	3	3	3	3	3	3	3	21
Pesqueira et al. (43)	Journal	3	3	3	3	3	3	3	21
Phan et al. (44)	Journal	3	3	3	3	3	3	3	21
Philip et al. (45)	Journal	3	3	3	3	3	3	3	21
Prasad et al. (46)	Journal	3	3	3	3	3	3	3	21
Rathore et al. (47)	Journal	3	3	3	3	3	3	3	21
Riswantini et al. (48)	Journal	3	3	3	3	3	3	3	21
Shahat Osman and Elragal (49)	Journal	3	3	3	3	3	3	3	21
Stevens et al. (50)	Journal	3	3	3	3	3	3	3	21
Takura et al. (51)	Journal	3	3	3	3	3	3	3	21
Thangarasu and Subramanian (52)	Journal	3	3	3	3	3	3	3	21
Tozzi et al. (53)	Journal	3	3	3	3	3	3	3	21
Tsui et al. (54)	Conference	3	3	3	3	3	3	3	21
Uddin Murad et al. (55)	Journal	3	3	3	3	3	3	3	21
Vicdan et al. (56)	Journal	3	3	3	3	3	3	3	21
Wang et al. (57)	Journal	3	3	3	3	3	3	3	21
Wu et al. (58)	Journal	3	3	3	3	3	3	3	21
Xie et al. (59)	Journal	3	3	3	3	3	3	3	21
Zakria et al. (60)	Journal	3	3	3	3	3	3	3	21
Zayoud et al. (61)	Journal	3	3	3	3	3	3	3	21
Zhou et al. (62)	Journal	3	3	3	3	3	3	3	21
Štufi et al. (63)	Journal	3	3	3	3	3	3	3	21
Kitchenham et al. (64)	Journal	3	3	0	3	3	3	3	18
Raja et al. (65)	Journal	3	0	3	3	3	3	3	18

This table is included as part of the methodological evidence of the review because it documents the application of the QA criteria, the scores assigned to each paper, and the basis for the final selection of studies. Its inclusion supports traceability, reproducibility, and direct verification of the quality assessment process.

As a result, out of the 65 papers evaluated, 63 met all the quality criteria, obtaining scores of 19 or higher. This rigorous assessment conclusively established the final number of publications included in the study.

### 2.5 Data extraction strategies

The data collected from each paper included detailed information such as the title, URL, source, year, country, ISSN, type of publication, publication name, authors, affiliation, quartile, H-index, citation count, methodology, abstract, and keywords. However, not all papers addressed all the research questions posed. To efficiently organize and manage this data, the Mendeley Desktop application was used. The outcomes of this process included: (1) a comprehensive bibliographic dataset containing standardized metadata for each study; (2) a set of scientometric indicators, such as database source, quartile classification, and citation metrics; (3) methodological descriptors summarizing the research focus, abstract, and keyword profile; and (4) an integrated and validated dataset ensuring transparency, traceability, and reproducibility in alignment with PRISMA 2020 and Kitchenham's methodological framework. All figures were regenerated and exported in high resolution to improve readability, avoid truncated labels, and ensure a clearer visual presentation of the systematic and bibliometric results.

## 3 Results and discussion

This section presents the outcomes derived from the literature search and selection process, which included a comprehensive review of articles published in journals and conferences specializing in the application of Big Data in the healthcare sector. Renowned databases were utilized, and the available studies were meticulously analyzed. Only the most relevant papers were selected, applying strict selection criteria and quality assessments to ensure the pertinence and suitability of the included publications. The following subsections detail the findings, offering a comprehensive view of the results obtained.

### 3.1 General description of studies

**Figure 3:**
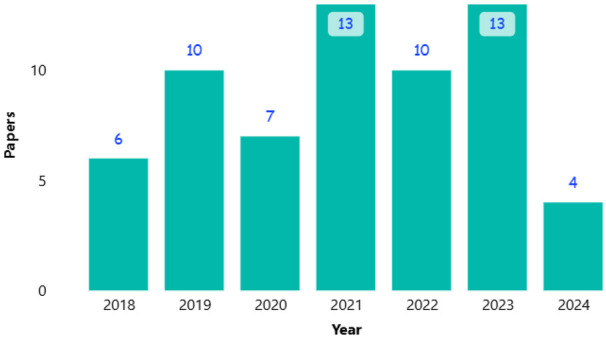
Distribution of papers by year.

Figure 3 illustrates the temporal distribution of the selected papers, organized by year of publication.

The distribution reveals a notable increase in research related to the topic between 2018 and 2021, peaking at 13 publications in 2021. This level persisted in 2023, suggesting renewed interest or thematic consolidation during these years. The lower number of papers observed in 2024 should be interpreted with caution because the search was completed on December 18, 2024, and some recently published papers may not yet have been fully indexed or accumulated sufficient visibility. Therefore, the 2024 data should not be interpreted as evidence of a real decline in research interest, but rather as a limitation associated with the temporal cutoff, indexing delay, and shorter citation exposure window. In 2019 and 2022, an intermediate production average of 10 publications was recorded, reflecting stability in research interest. These data indicate growth and the establishment of relevance for this topic in key years.

In comparison with previous studies, the trends in publication show similarities and differences. For instance (67), identified peaks in 2012 and 2016, followed by a significant decline in 2018. Other studies, such as (72), observed a consistent increase in the number of papers from 2018 to 2022. Similarly (75), highlighted notable increases between 2015 and 2016 and 2017–2018, with additional growth in 2020 and 2021. Recent research, such as (76) and (77), also reported growth in 2022.

The findings suggest that years with high production peaks, such as 2021 and 2023, are pivotal periods for consolidating trends or significant advancements in the field. This underscores the importance of identifying specific events or technological developments that drove these publications. Conversely, the decline in 2024 may represent an opportunity to redirect research efforts toward strengthening existing areas. Finally, the stability observed between 2019 and 2022 reinforces the need to evaluate the continuity of key topics addressed during those periods.

**Figure 4:**
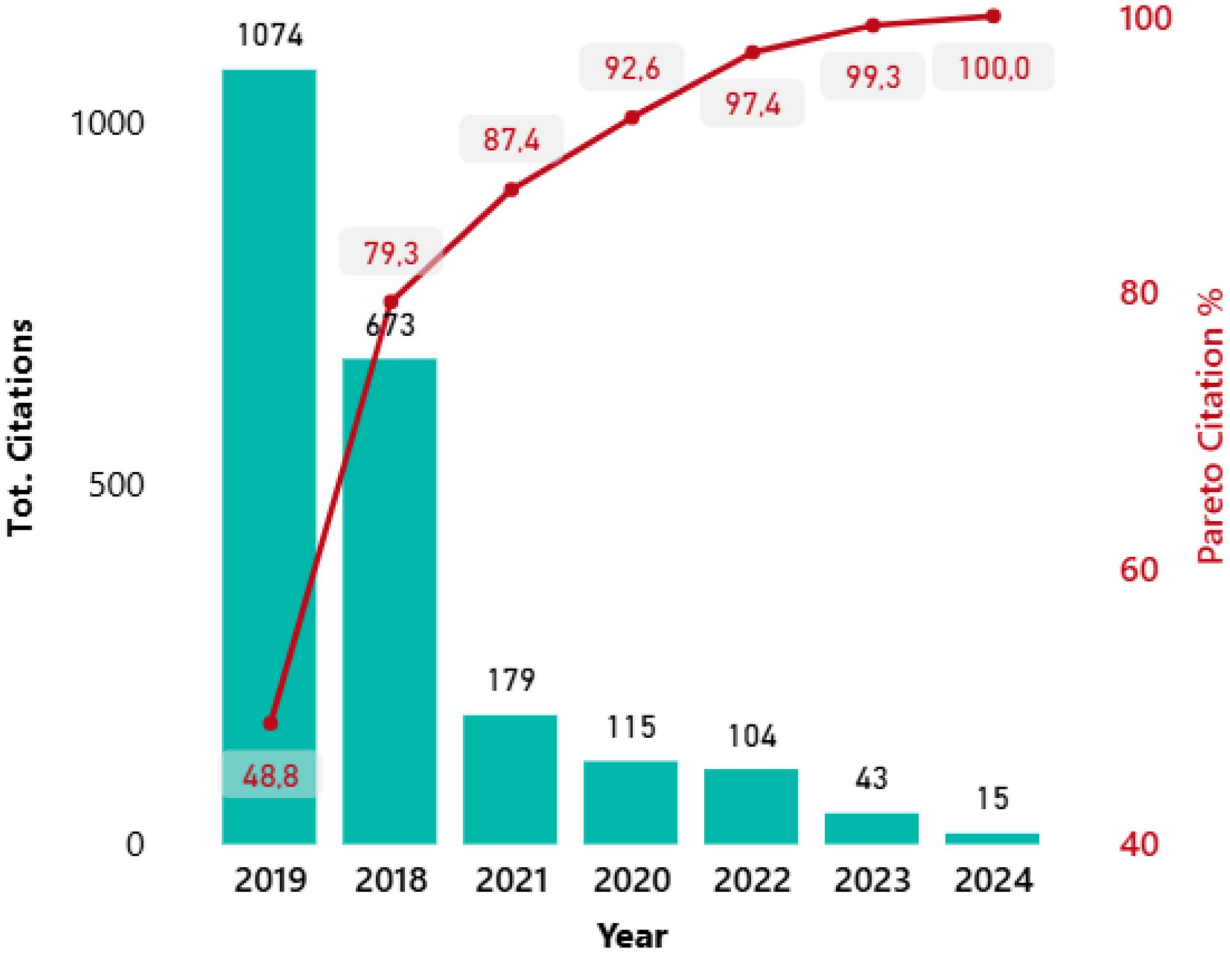
Pareto chart of citations by year.

Figure 4 presents a Pareto chart of citations by year, enabling the identification of periods that concentrate the highest scholarly impact. Its inclusion is justified as it reveals temporal asymmetries in citation patterns and provides insight into the maturation dynamics of the research field.

As observed in the results, citations are strongly concentrated in the earliest years of the analyzed period. Papers published in 2019 account for 1,074 citations, representing 48.8% of the total, while 2018 and 2019 together accumulate 79.3% of all citations. This pattern reflects a typical citation-age effect, where older publications have had more time to accumulate scholarly visibility and become reference points for subsequent studies.

The lower citation contribution of papers published from 2020 onward should therefore be interpreted cautiously. It does not necessarily indicate lower relevance, but rather shorter exposure time, progressive thematic diversification, and the natural delay between publication and citation accumulation. Consequently, the Pareto pattern suggests that the field has been shaped by a limited group of early influential publications, while more recent studies may still be consolidating their academic impact.

**Figure 5:**
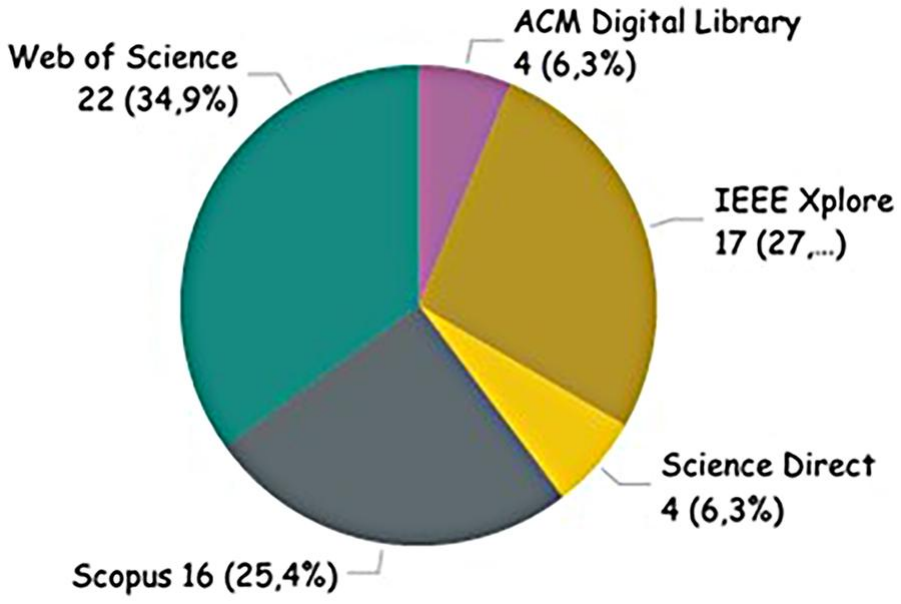
Distribution of papers by source.

Figure 5 summarizes the distribution of included papers by bibliographic source, while Table 4 complements it by reporting citations, papers, and citations per paper for each source. This joint presentation is justified because it simultaneously captures coverage (volume of papers) and impact (citation concentration), enabling a more robust assessment of source dominance and potential selection effects.

**Table 4:** Detailed breakdown of citations and papers by source.

Source	Citat./Paper	N° Citations	N° Papers
Web of Science	47	1,036	22
Science Direct	43	171	4
Scopus	34	544	16
IEEE Xplore	22	382	17
ACM Digital Library	18	70	4
Total	35	2,203	63

As observed in the results, Web of Science contains the largest number of papers and the highest citation volume, with 22 papers and 1,036 citations. This suggests that a substantial part of the most visible research on Big Data in healthcare is concentrated in highly indexed journals. Scopus also shows a balanced contribution, with 16 papers and 544 citations, while IEEE Xplore contributes 17 papers but with a lower citation density, which may reflect its stronger orientation toward engineering and conference-based publication channels. ScienceDirect and ACM Digital Library contain fewer papers, but their inclusion contributes to the diversity of the corpus by capturing studies from complementary publication ecosystems. Overall, the source distribution confirms the need to use multiple databases in systematic and bibliometric reviews, since each source contributes differently in terms of coverage, disciplinary orientation, and citation visibility.

In comparison, studies such as (72) highlight that IEEE Xplore hosts the highest number of papers, followed by Elsevier, Springer, and Taylor & Francis Online. Meanwhile (76), emphasizes a high presence of papers in Google Scholar, IEEE Xplore, IOPScience, and Scopus, with 15 publications each. Both studies underscore the relevance of IEEE Xplore in disseminating research on Big Data in healthcare.

For managerial and research practice, the observed source asymmetries recommend triangulating evidence across databases when translating insights to other sectors (e.g., finance, logistics, manufacturing), as database bias may overrepresent certain publication cultures and undercapture practitioner-facing venues. Geographically, extending the search strategy to regionally strong indexers and non-English corpora can improve representativeness for emerging economies and underindexed regions, where impactful work may not be fully reflected in WoS-dominant pipelines. Longitudinally, repeating the same sourcing protocol across different time windows can reveal whether citation concentration persists or shifts, supporting more resilient evidence synthesis and better forecasting of future “high-impact” outlets.

Table 5 synthesizes the key findings of the 63 studies analyzed, grouped by their predominant methodological category. This organization allows the identification of recurrent patterns in methods, datasets, evaluation metrics, and limitations, facilitating cross-comparison and the extraction of general lessons. Only studies explicitly aligned with the objectives of this review were included, prioritizing: (i) clear relevance to healthcare transformation through big data, with a focus on clinical decision-making, patient data management, or healthcare analytics; (ii) methodological robustness and the reporting of comparable metrics (e.g., accuracy, AUC, BLEU, RMSE) along with reproducible validation protocols (cross-validation, external validation, or large-scale simulations) and sufficient data description; (iii) dataset traceability and transparency regarding data sources; and (iv) consistency of results across settings. Studies lacking interpretable or commensurable metrics were excluded, while traceability was maintained through the reference identifiers listed under each category.

**Table 5:** Synthesis of findings by methodological category.

Method category	Methods used	Datasets	Performance	Limitations	Refs.	Qty. (%)
*Deep learning—NLP/dialog (Seq2Seq, attention)*	LSTM/GRU Seq2Seq with Attention; Bag-of-Words; Beam Search; BLEU evaluation	Dialog/text corpora (threat/coercion persuasion tasks)	Consistent BLEU-4 ≈ 0.8537 across studies	Focus on “threat” as primary motivator; limited usability/UX; restricted generalizability	(6, 19, 35, 46, 51, 53, 56)	7 (14.3)
*Frameworks & prototypes (IoT/IoHT/Conceptual)*	IoHT/IoT fog–cloud architectures; BDA conceptual frameworks; framework/prototype development	Unspecified or limited validation; patient records; user reviews	Reduced latency/packet drop; improved quality/management; mAP 79% in KMS	Heterogeneous data complexity; real-time integration challenges; limited validation in diverse ntexts	(4, 10, 12, 44, 49, 54, 62)	7 (14.3)
*Big data platforms & architectures*	Hadoop/MapReduce/Spark; BDA platforms; intelligent processing pipelines	WBAN sensor data; IHIS (anonymized); heterogeneous medical big data	TPC-H compliant; ingestion of 1–3 TB in 2.94–5.99 h; Spark processed 100 TB in 23 m vs. 71 m with doop	Slow adoption; infrastructure and skills gap; local compliance/regulatory barriers	(1, 20, 37, 40, 47, 52)	6 (12.2)
*Machine learning & statistical*	k-means, Decision Trees, SVM (MapReduce), Naïve Bayes, statistical analyses (ANOVA, mAP, RMSE)	COVID-19 cases, cardiopathies, NHANES, perinatal 250 × 60, healthcare fraud datasets	Accuracy ranges 72%–92%+; reduced processing time with MapReduce	Data quality/completeness issues; demographic representativeness; limited sample sizes	(7, 8, 16, 41, 45, 58)	6 (12.2)
*Deep learning—clinical/imaging/prediction*	CNN/RNN/LSTM; Autoencoders; Bayesian DL for feature selection; DQN/RL for diagnosis/triage	COVID-19 tweets (health NLP), EMRs, UCI Breast Cancer/Statlog, KNHNES, hospital ECGs	High performance (e.g., 95.8% precision; 99.8% ACC; solid AUC/recall)	Dependence on specific/imbalanced datasets; sampling biases; computational complexity	(3, 18, 25, 28, 34)	5 (10.2)
*Structural modeling & adoption (SEM/PLS)*	PLS-SEM/SEM (SmartPLS3); modeling of BDAC capabilities and RM/GSCM practices	Surveys of healthcare professionals (Italy, Malaysia, Egypt)	Significant BDAC effects on QoHS/STR; full mediations confirmed	Country-specific context; limited external validity; underexplored tangible indicators	(14, 15, 23, 24, 33)	5 (10.2)
*Decision & policy methods (DEMATEL/Delphi/TAM/MCDM)*	DEMATEL; Policy Delphi; Fuzzy BWM/VIKOR; TAM/MTAM with SmartPLS	Expert opinions; 193 arguments; adoption surveys	Identification of 11 BDAC capabilities; 91% consensus; PEoU → PU; BI dependent on PU	Context-specific findings; social/ethical uncertainty; resource constraints limiting adoption	(31, 36, 55, 60)	4 (8.2)
*Legal/policy/qualitative studies*	Comparative legal analysis; qualitative interviews; PPP models with predictive analytics	Legal materials; 12 European pilots; financial scenarios	Identification of regulatory gaps; valuable qualitative insights	Limited empirical evidence; insufficient governance and data protection frameworks	(27, 39, 50)	3 (6.1)
*Optimization & OR algorithms*	MILP; metaheuristics (Hybrid Dingo Coyote, PSO); PHA with network coding/LDPC	Framingham, HAR/mHealth; channel simulations	Improved SINR/WSR; +13%–17% over classical baselines; reduced latency/errors	Domain specificity; network assumptions/parameter dependencies; uncertain transferability	(9, 26, 38)	3 (6.1)
*Security/privacy/ethics*	Surveys and comparative analysis of anonymization/encryption; ethical compliance; TPC-H tests	Unspecified; anonymized IHIS	Evidence of systemic gaps (320% ↑ in 2016); compliant platforms	Insufficient technical support; reactive approaches; localized regulatory scope	(2, 30, 63)	3 (6.1)

#### 3.1.1 Method category

The taxonomy exposes two dominant, mid-maturity cores, DL–NLP/Dialog and IoT/IoHT Frameworks & Prototypes (each 14.3%), coexisting with infrastructural work (Big-Data Platforms, 12.2%) and classical ML/Statistical pipelines (12.2%). A second tier, Clinical/Imaging DL and SEM/PLS adoption models (10.2% each), signals translation toward clinical and managerial value. Policy-oriented segments (DEMATEL/Delphi/TAM, 8.2%; legal/qualitative, 6.1%) and Security/Privacy/Ethics (6.1%) indicate that obstacles are socio-technical, not merely algorithmic. Optimization/OR (6.1%) appears as a complementary stream for operational efficiency.

#### 3.1.2 Methods used

Methodological emphasis spans Seq2Seq with attention and BLEU evaluation for dialog tasks; CNN/RNN/LSTM variants (including autoencoders and DQN/RL) for clinical prediction; and classical pipelines (trees, SVM, Naïve Bayes, k-means) grounded in inferential statistics (ANOVA, mAP, RMSE). System-level studies leverage Hadoop/MapReduce/Spark and IoT/IoHT architectural frameworks; organizational evidence is modeled with PLS-SEM/SEM, DEMATEL, Delphi, Fuzzy BWM/VIKOR, TAM/MTAM. The mix indicates a field where algorithmic sophistication advances in parallel with explanatory and decision frameworks required for deployment.

#### 3.1.3 Datasets

Data usage is heterogeneous and often corpus-specific: dialog text for NLP (threat/coercion), EMRs, UCI Breast Cancer/Statlog, KNHNES, hospital ECG, and WBAN/IHIS for platform benchmarking; many prototypes report unspecified or narrowly validated sets. Adoption studies rely on surveys of healthcare professionals (Italy, Malaysia, Egypt), whereas policy analyses use expert-opinion panels. The pattern reflects a bias for availability (well-curated or single-country data) and highlights a deficit of standardized, multi-institutional datasets for external validation.

#### 3.1.4 Performance

Algorithmic studies report high accuracy (e.g., precision 95.8%, ACC 99.8%, BLEU ≈ 0.8537), processing-time gains with MR/Spark (100 TB in 23 min), and SINR/WSR improvements in optimization; IoT/IoHT prototypes show reduced latency/packet loss and a mAP of 79% in knowledge-management systems. Yet cross-study comparability is limited by heterogeneous metrics, missing AUC/CIs in several reports, and evaluations under controlled conditions or small samples, constraining in-production generalizability.

#### 3.1.5 Limitations

Recurrent constraints include restricted generalization from specific/imbalanced datasets, small samples and labeling bias; real-time integration and interoperability gaps (EHR/IoT); computational burden and resource scarcity; and weak governance, privacy/security, and metric standardization. In dialog NLP, a narrow threat-focus limits clinical relevance and usability. Adoption evidence often suffers context dependence (country-specific), while platform studies face infrastructure and skills deficits.

#### 3.1.6 N° studies (%)

The distribution is balanced yet fragmented: four categories account for ∼51% of studies (DL–NLP/Dialog and Frameworks/Prototypes at 14.3% each; Platforms and ML/Statistical at 12.2% each). The remaining 49% spans clinical application, organizational adoption, policy/decision, legal/qualitative, optimization, and security. This dispersion portrays a multi-layered field in which technical progress is not consistently matched by deployment, compliance, and external-evaluation maturity.

To strengthen clinical transferability, multi-center, standardized datasets with consistent reporting (AUC, F1, CIs) are imperative. Platform maturity must be tied to operational clinical cases with SLO/latency targets and TCO analyses, bridging benchmarks and production. Security, privacy, and interoperability (e.g., HL7/FHIR, consent, auditability) should be first-class design constraints, not afterthoughts. Embed XAI and organizational modeling (SEM/PLS, DEMATEL) across the lifecycle to accelerate responsible adoption. Prioritize impact evaluations quantifying effects of BDA/ML on care quality, process efficiency, and economics to evidence real-world value.

### 3.2 Research questions answers

Below are the answers to the research questions posed in this study, supported by the analyses, discussions, and insights derived from the comprehensive review of the selected literature. Key implications for future research are also included, providing a solid framework for advancing this field of study.

#### 3.2.1 RQ1: what indicators and parameters are used to evaluate the effectiveness of big data usage?

**Figure 6:**
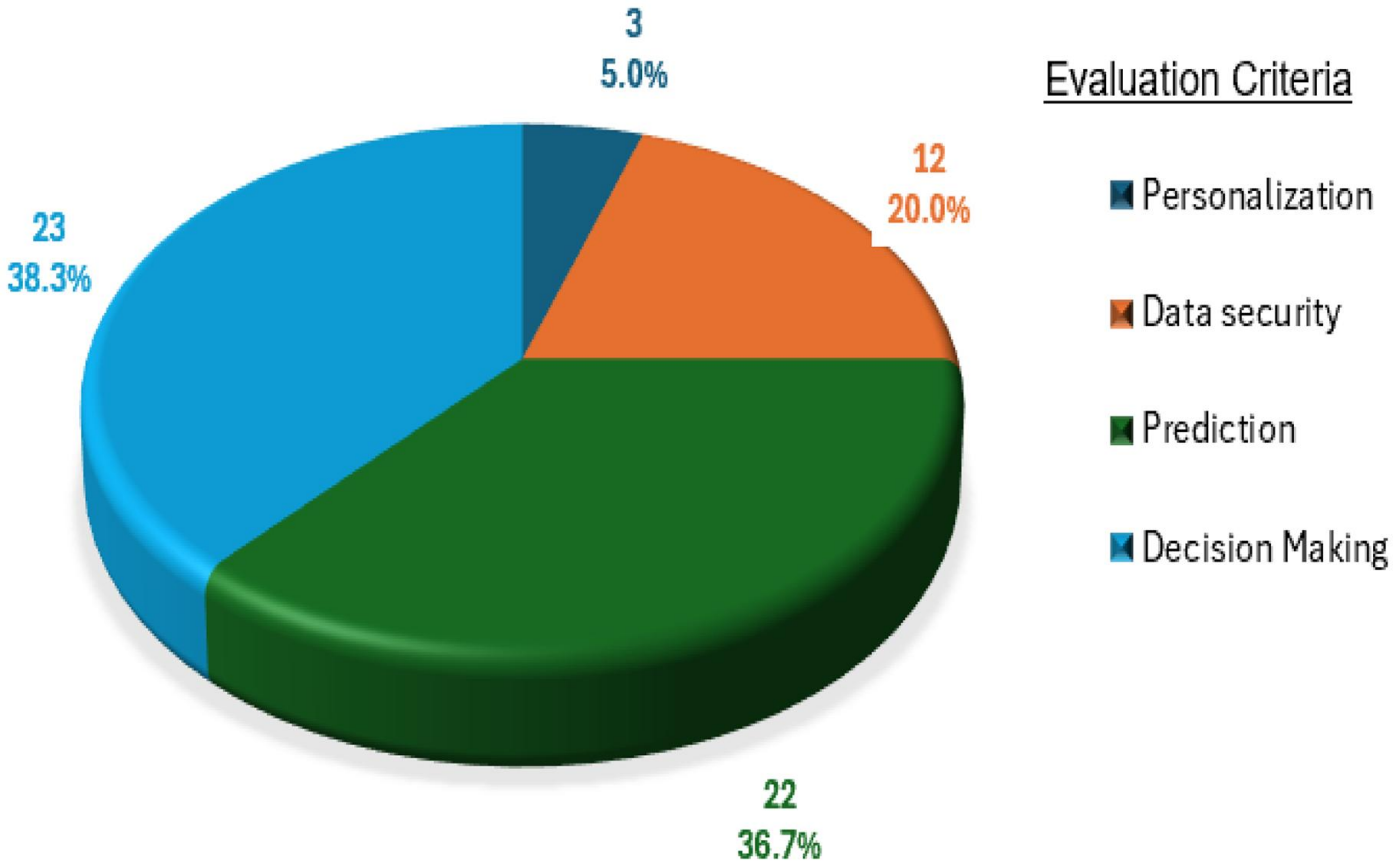
Number of papers by evaluation criterion.

Table 6 and Figure 6 present the criteria employed to evaluate the effectiveness of Big Data, divided into four main categories. Table 7 provides the analytical detail of the classification, including the references associated with each criterion and their corresponding frequencies. Figure 6 complements this information by offering a consolidated graphical representation of the proportional distribution of these criteria, allowing a faster visual comparison of the dominant dimensions. Therefore, both formats are retained because they provide complementary perspectives: the table ensures precision and traceability, while the pie chart facilitates the visual interpretation of the overall distribution.

**Table 6:** Effectiveness criteria of big data.

Criterion	Reference	Quantity (%)
Personalization	(21, 34, 56)	3 (5.0)
Data security	(2, 4, 17, 22, 24, 27, 29, 30, 32, 37, 57, 63)	12 (20.0)
Prediction	(1, 3, 5, 8, 11, 12, 18, 23, 26, 36, 40, 41, 44, 45, 48, 51, 53, 54, 59–62)	22 (36.7)
Decision Making	(6, 7, 10, 13, 16, 19, 20, 25, 28, 31, 33, 35, 38, 39, 42, 43, 46, 47, 49, 50, 52, 55, 58)	23 (38.3)

**Table 7:** Quartiles by publication source.

Source	Q1	Q2	Q3	SQ	Total
Web of Science	12	8	0	2	22
IEEE Xplore	17	0	0	0	17
Scopus	8	5	2	1	16
ACM Digital Library	3	1	0	0	4
Science Direct	3	0	0	1	4
Total	43	14	2	4	63

To classify the effectiveness criteria, each paper was coded according to its dominant analytical focus. The four categories—personalization, data security, prediction, and decision-making—were not treated as mutually exclusive representations of the entire content of each study, since several papers may address more than one dimension. Instead, each paper was assigned to the category that best reflected its main objective, methodological orientation, reported outcomes, and discussion. When a paper covered multiple criteria, the dominant criterion was selected to avoid double counting and to maintain consistency in the quantitative synthesis. Therefore, the classification presented in Table 7 should be interpreted as a structured synthesis of the primary orientation of the studies, rather than as an exhaustive coding of all possible effectiveness dimensions addressed in each paper.

Most studies (38.3%) focus on decision-making as the primary criterion, emphasizing the importance of Big Data in improving administrative and clinical processes. Prediction also plays a significant role (36.7%), highlighting its relevance in anticipating events and risks across various contexts. Data security, accounting for 20%, emerges as a key concern in implementing Big Data technologies. Lastly, personalization, although less represented (5%), demonstrates its emerging importance in individualizing services based on data analysis. These results suggest a balance between technical and strategic criteria, with a clear emphasis on practical applications.

In comparison (66), identifies factors such as uncertainty, imprecision, ambiguity, vagueness, and incompleteness as critical to the effectiveness of Big Data Analytics (BDA) in healthcare organizations. These factors highlight how deficiencies in data quality can negatively impact decision-making and compromise the accuracy and usefulness of predictions. Additionally, inherent data limitations affecting the effectiveness of analytical tools are addressed, contrasting with the predominant focus on prediction and decision-making.

These results indicate that the effectiveness of Big Data in healthcare is evaluated mainly through its capacity to improve decisions, anticipate risks, and strengthen secure data management. Compared with previous studies, this emphasis is consistent with the relevance of uncertainty, data quality, and analytical reliability in healthcare organizations.

#### 3.2.2 RQ2: what quartile rankings do the journals publishing research on the impact of big data in healthcare hold?

**Figure 7:**
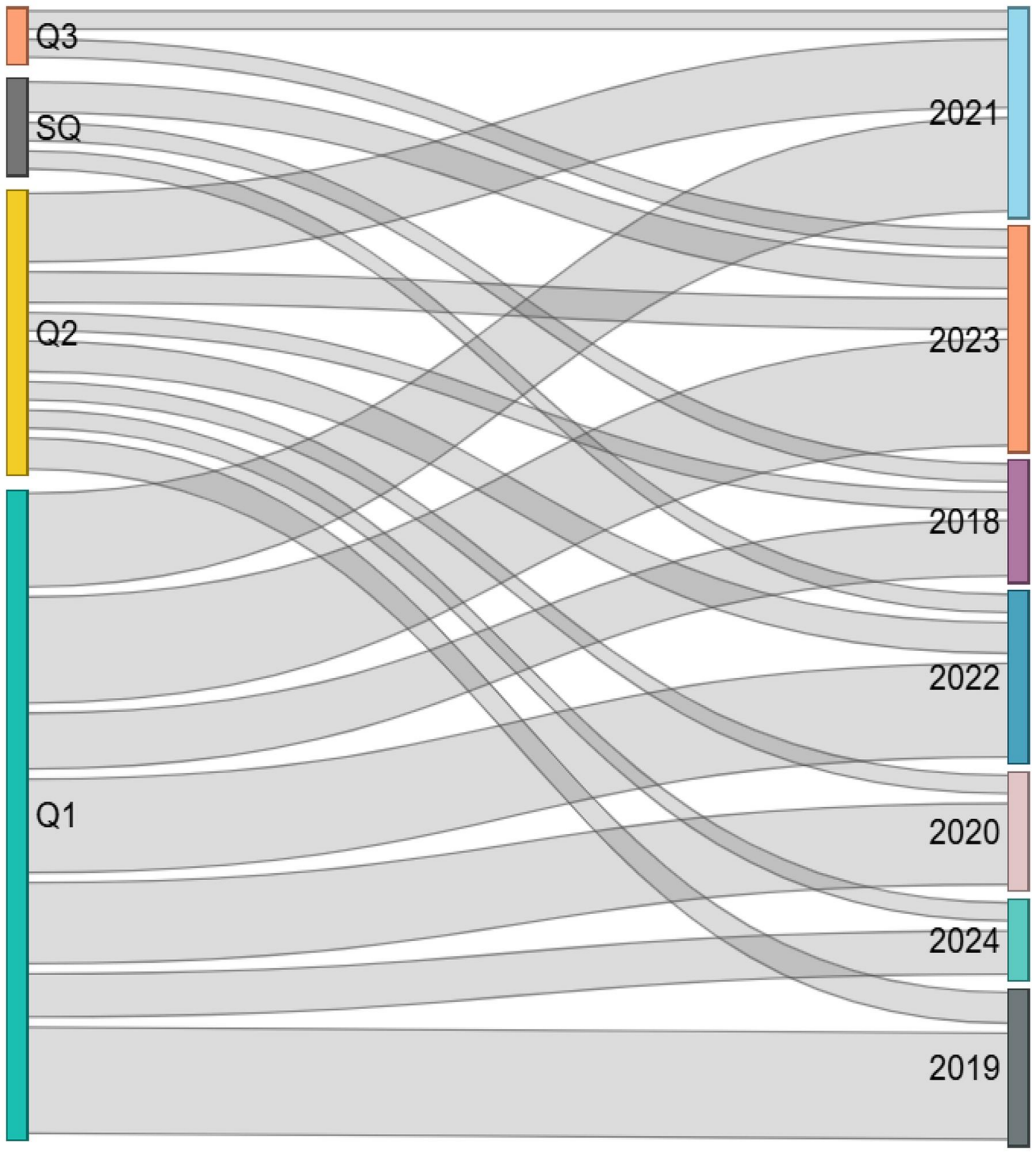
Quartiles by publication year.

Figure 7 and Table 7 present complementary perspectives on the quartile distribution of the selected papers. Figure 7 shows the evolution of quartile categories by year, allowing the temporal behavior of Q1, Q2, Q3, and SQ publications to be observed across the study period. Table 8, in contrast, provides the detailed distribution of quartile categories by publication source, enabling the identification of which databases concentrate the largest number of high-impact papers.

**Table 8:** Most frequently used concepts (bigrams) by year.

Bigram	2018	2019	2020	2021	2022	2023	2024	Total
Big data	5	5	4	5	4	9	1	**33**
Data analytics	2	2	2	2	1	1	1	**11**
Healthcare industry	1	2	0	1	1	0	0	**5**
Healthcare sector	0	0	0	1	1	2	0	**4**
Machine learning	1	0	2	0	0	1	0	**4**
Data analysis	0	0	1	0	1	1	0	**3**
Healthcare data	1	0	0	0	0	2	0	**3**
Healthcare organizations	1	0	0	1	0	0	1	**3**
Data technology	0	0	1	1	0	0	0	**2**
Healthcare services	0	0	0	0	0	0	2	**2**
Learning models	0	0	1	0	0	1	0	**2**
Acquisition methods	0	0	1	0	0	0	0	**1**
Addressing bda	0	0	0	1	0	0	0	**1**
Adherence score	0	0	0	1	0	0	0	**1**
Affectively engage	0	0	0	0	1	0	0	**1**
…	…	…	…	…	…	…	…	**…**
Total	34	25	34	61	36	62	16	268

Bold values indicate the total frequency of each bigram across all analyzed years.

Most papers are published in Q1 journals, representing 68.3% of the corpus, followed by Q2 journals with 22.2%. This distribution indicates that research on Big Data in healthcare is mainly concentrated in high-impact publication venues. Regarding the temporal behavior shown in Figure 7, Q1 publications appear consistently across the study period, with stronger concentration in the years with higher scientific production, particularly 2021 and 2023. Q2 publications also appear across several years but with lower intensity, while Q3 and SQ categories are sporadic and marginal. At the source level, Table 8 shows that IEEE Xplore and Web of Science account for a large proportion of Q1 and Q2 studies, whereas Q3 and SQ publications have limited representation.

In their research (76), highlights the significance of quartiles, noting that Q1 journals are regarded as the most prestigious and are frequently cited in the scientific community. Similarly (71), emphasizes that inclusion in Q1 is an indicator of the high quality and academic rigor of journals, which enhances the prestige of the studies cited.

This pattern suggests that the field has achieved strong visibility in indexed and high-quality journals. In line with previous studies, the predominance of Q1 and Q2 sources reinforces the academic relevance of Big Data research in healthcare and supports the reliability of the evidence base analyzed in this review.

#### 3.2.3 RQ3: what predominant concepts have been used in research on big data and its impact on healthcare?

Table 8 presents the predominant concepts (bigrams) used in research on Big Data and its impact on healthcare, organized by year. This data provides insight into thematic trends and key areas of focus in scientific literature, offering a detailed view of the field's development.

The most frequent bigram is “big data”, with 33 mentions, highlighting its centrality in research over the years analyzed. “data analytics” ranks second with 11 mentions, emphasizing data analysis as a critical tool. Other concepts, such as “healthcare industry” and “machine learning”, reflect a focus on practical applications and advanced methodologies, although they appear less frequently. The increase in mentions in 2021 and 2023 indicates significant growth in academic output during these years. Meanwhile, less frequent bigrams, such as “adherence score”, suggest emerging areas within the field.

In contrast, terms such as “AI” and “machine learning”, particularly in their application to healthcare and Big Data Analytics, are gaining relevance in current scientific research.

According to (76), the use of these terms has grown significantly, reflecting their increasing importance in academia. This growth underscores how advanced technologies are influencing research and development at the intersection of Big Data and healthcare.

The temporal distribution of bigrams shows that the topic has remained stable across the analyzed period, although specific concepts vary according to technological developments and healthcare priorities. Overall, the results show a field structured around data analytics, machine learning, healthcare management, and digital transformation.

#### 3.2.4 RQ4: what key terms tend to co-occur in studies on big data and its impact on healthcare?

**Figure 8:**
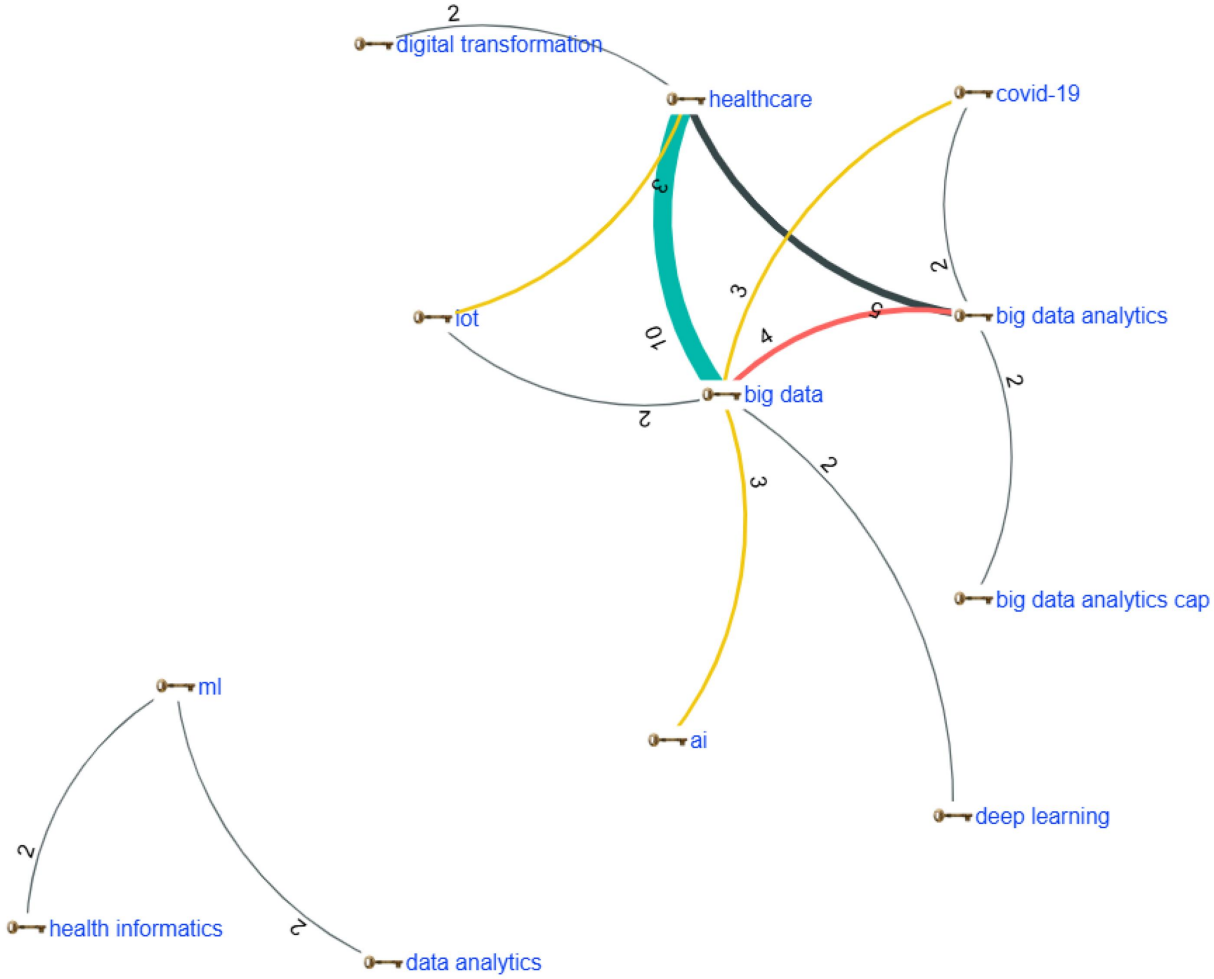
Bibliometric network of keywords with the highest co-occurrence.

Figure 8 visualizes the co-occurrence of key terms in research on Big Data and its impact on healthcare. This analysis helps identify relevant thematic connections, focus areas, and potential intersections among key concepts.

To construct the keyword co-occurrence network, the bibliometric analysis was performed using RAj (Research Assistant j), a research support tool developed by Dr. Javier Gamboa for systematic and bibliometric review processes. The analysis computed the association strength between terms to reveal conceptual coupling in Big Data-healthcare literature. Candidate terms were derived from author keywords and normalized title/abstract n-grams, including unigrams and bigrams, after lower-casing, tokenization, lemmatization, and domain stop-word removal. A term-document matrix **W** **=** **[w_t,d_]** was then constructed using binary or TF-IDF weights. Co-occurrence was defined as the joint presence of terms within the same document; the association strength between terms was quantified using cosine similarity of their document vectors:

$$cos(t_{i},t_{j})=\frac{\sum_{d=1}^{m}\;\;w_{i,d}w_{j,d}}{\sqrt{\sum_{d=1}^{m}\;{\;w}_{i,d}^{2}}\;\sqrt{\sum_{d=1}^{m}\;{\;w}_{j,d}^{2}}}$$

Where $w_{i,d}$ is the weight of term $t_{i}$ in document d. Higher $cos(t_{i},t_{j})$ values indicate stronger co-occurrence and conceptual affinity between terms. The resulting weighted graph was thresholded using minimum term frequency and similarity criteria and then pruned to its giant component. Communities were detected to identify thematic clusters within the corpus. This procedure provided a robust and scale-independent map of key co-occurring terms, answering RQ4 with greater methodological transparency and reproducibility. To improve readability, the keyword network was generated in high resolution, with adjusted label positioning and node distribution to reduce overlap and avoid truncated labels.

The keyword network confirms that “big data” occupies a central position and is strongly connected with terms such as “healthcare,” “big data analytics,” “covid-19,” “IoT,” “digital transformation,” and “deep learning.” These relationships indicate that Big Data research in healthcare is organized around analytical capacity, emerging technologies, and data-driven responses to health challenges.

According to Reshi et al. (69), the terms “big data” and “healthcare” are the most co-occurring keywords in the selected research articles, emphasizing their relevance in the topics addressed. Similarly (71), highlights the frequency of their joint appearance, underscoring their importance in the current scientific literature. Furthermore (77), reinforces this observation, noting that the combination of “big data” and “healthcare” is essential for data analysis in the health sector.

The prominence of concepts like “big data” and “covid-19” indicates opportunities to explore how these technologies can enhance healthcare during critical public health situations. Additionally, connections with “IoT” and “deep learning” suggest promising areas for future interdisciplinary research. These findings are also applicable in sectors such as digital health and crisis management, fostering data-driven strategies.

#### 3.2.5 RQ5: what main thematic categories are addressed in research on big data and its impact on healthcare?

**Figure 9:**
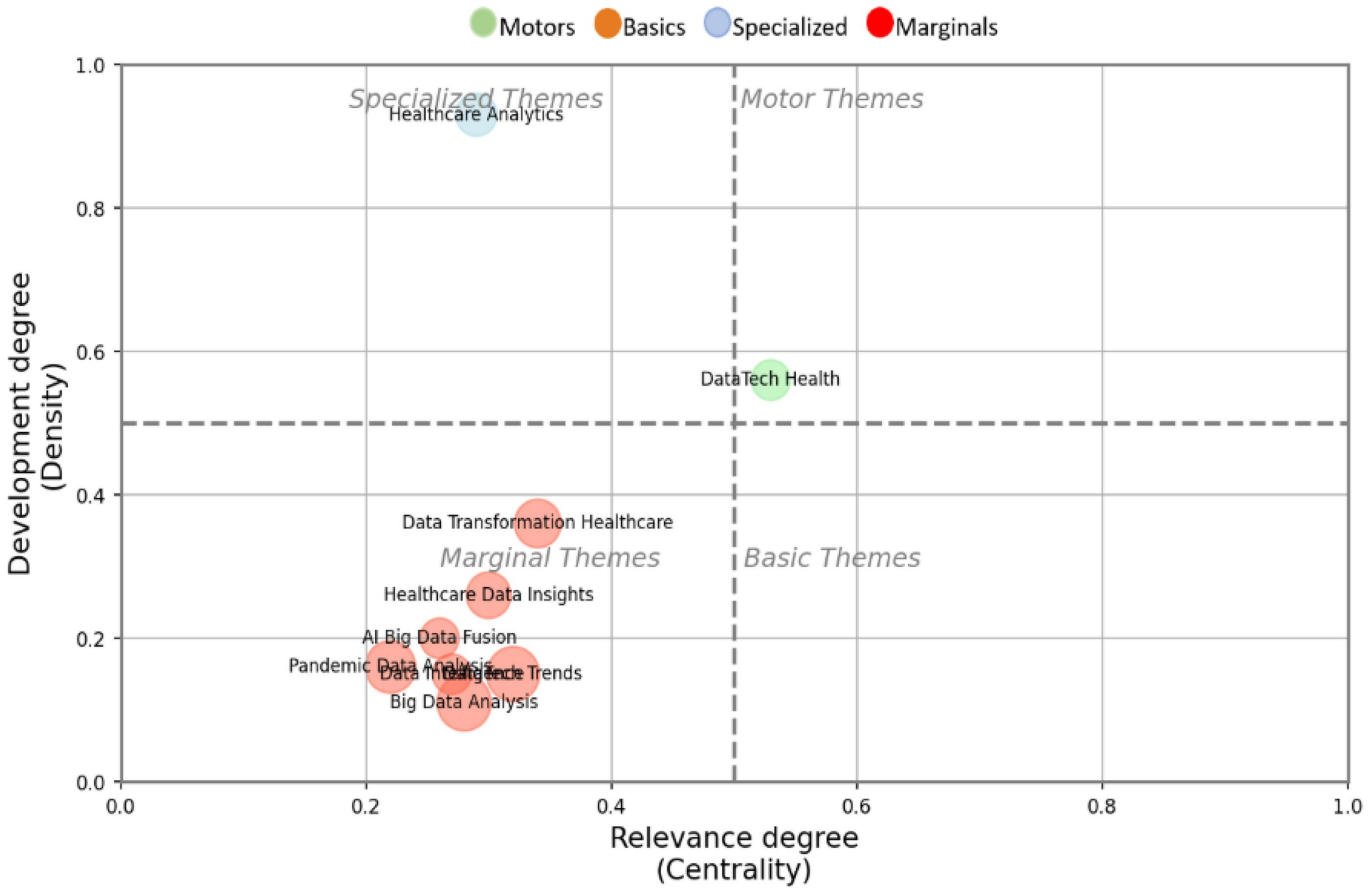
Thematic map of categories.

Figure 9 and Table 9 classify the thematic categories in research on Big Data and its impact on healthcare, evaluating their relevance (centrality) and degree of development (density). These parameters identify motor, specialized, basic, and marginal themes, providing a thematic map of the field.

**Table 9:** Thematic categories.

Topic	Density	Centrality	Total quotes	Total documents	Category
Healthcare analytics	0.93	0.29	1,246	19	Specialized
DataTech health	0.56	0.53	1,102	13	Motor
Data transformation healthcare	0.36	0.34	1,591	16	Marginal
Healthcare data insights	0.26	0.30	1,456	27	Marginal
AI Big data fusion	0.20	0.26	330	12	Marginal
Pandemic data analysis	0.16	0.22	558	8	Marginal
Data intelligence	0.15	0.32	766	14	Marginal
DataTech trends	0.15	0.27	330	12	Marginal
Big data analysis	0.11	0.28	802	16	Marginal

As shown in the results, Healthcare Analytics exhibits the highest centrality (0.93) with 1,246 occurrences, confirming its integrative role; its moderate density (0.29) suggests rapid expansion driven by the need for evidence-based clinical decision-making. According to the findings presented, DataTech Health combines strong centrality (0.56) and density (0.53) with 1,102 records, positioning it as a motor theme and reflecting sustained investments in digital health infrastructures. Based on the observed distribution, Data Transformation Healthcare reports the largest volume (1,591 occurrences; 0.36; 0.34) but lower conceptual cohesion, indicating theoretical fragmentation typical of early-stage digital transformation processes. Similarly, Healthcare Data Insights accumulates 1,456 occurrences with limited centrality (0.26), suggesting intensive operational use rather than full theoretical consolidation. Finally, themes such as AI Big Data Fusion (330; 0.20; 0.26) and Pandemic Data Analysis (558; 0.16; 0.22) remain peripheral, likely constrained by regulatory challenges and data heterogeneity.

The predominance of marginal themes in the thematic map does not indicate that these topics lack relevance. Rather, it suggests that several research lines in Big Data and healthcare remain weakly connected to the central conceptual structure of the field and have not yet reached strong internal consolidation. In this context, marginality reflects low centrality and limited density, which means that these topics are still fragmented, emerging, or insufficiently integrated with the dominant research streams. This pattern may be explained by the heterogeneity of Big Data applications in healthcare, the diversity of data sources and analytical methods, and persistent challenges related to interoperability, data governance, privacy, and clinical validation. Therefore, the thematic map suggests that the field is still in a process of maturation, where consolidated areas such as Healthcare Analytics and DataTech Health coexist with emerging but less integrated topics such as AI Big Data Fusion, Pandemic Data Analysis, and Healthcare Data Insights. This finding is relevant because it identifies opportunities for future research to strengthen conceptual links, develop more standardized methodologies, and move currently marginal themes toward more central and mature positions within the field.

According to Reshi et al. (69), specialized and basic categories are the most prominent in Big Data research in healthcare, due to their focus on concrete health problems. Similarly (78), emphasizes the importance of basic categories, as they provide the theoretical and methodological foundations essential for understanding the overall impact of this technology.

The dominance of advanced analytics indicates strong transferability to sectors such as finance, logistics, and smart governance, where data exploitation can generate competitive advantages. Regions with lower technological maturity may accelerate adoption by prioritizing interoperable architectures and robust data governance. In the medium term, AI–Big Data convergence is expected to shift currently marginal themes toward central positions, reshaping organizational strategies across industries, geographic contexts, and time horizons.

## 4 Conclusion and future research

The identified indicators, such as decision-making and prediction, have proven to be the most prominent criteria for evaluating the effectiveness of Big Data usage in the healthcare sector, while personalization emerges as a developing dimension (RQ1). Additionally, research is concentrated in high-impact journals, particularly those classified as Q1, reflecting the quality of the scientific literature in this field (RQ2). Regarding predominant concepts, “big data analytics” leads as a key topic, alongside related terms such as “machine learning” and “healthcare industry,” showcasing the thematic diversity of this area (RQ3). Moreover, co-occurring keywords like “big data” and “IoT” highlight the integration of emerging technologies and healthcare applications (RQ4). Finally, thematic categories are led by consolidated areas such as Healthcare Analytics and the motor theme DataTech Health, while high-volume but less cohesive topics like Data Transformation Healthcare and emerging areas including AI Big Data Fusion and Pandemic Data Analysis reveal a balance between mature research lines and evolving opportunities (RQ5).

Based on these findings, several concrete recommendations are proposed. First, healthcare institutions should prioritize interoperable data infrastructures that integrate electronic health records, IoT-based monitoring systems, clinical repositories, and administrative databases. Second, future studies should apply standardized evaluation metrics, such as accuracy, sensitivity, specificity, AUC, *F*1-score, processing time, and cost-effectiveness indicators, to improve comparability across Big Data applications. Third, predictive models should be validated using multicenter and heterogeneous datasets to strengthen external validity and reduce context-specific bias. Fourth, data governance, privacy, cybersecurity, and ethical compliance should be incorporated from the design stage of Big Data projects. Fifth, explainable AI approaches should be integrated into Big Data-based decision-support systems to improve transparency, clinical trust, and accountability. Finally, future research should move beyond technical performance and evaluate the real impact of Big Data solutions on healthcare quality, patient outcomes, operational efficiency, and evidence-based decision-making.

This study faced several limitations. First, although the search strategy included major scientific databases such as Web of Science, IEEE Xplore, Scopus, ScienceDirect, and ACM Digital Library, PubMed was not included. This may have limited the retrieval of biomedical and clinical studies specifically indexed in that database. Second, the bibliographic search covered studies published up to 2024; therefore, more recent papers published or indexed after the search cutoff may not have been captured, which is relevant in a rapidly evolving field such as Big Data in healthcare. Third, the review identified a lack of studies with directly comparable methodologies, datasets, and evaluation metrics, which limits cross-study comparison. Future research should expand the search strategy to include PubMed and other specialized biomedical databases, update the evidence base with post-2024 studies, and further explore emerging areas such as Big Data integration with IoT, public health surveillance, personalized healthcare, and explainable AI-based decision-support systems.

## Data Availability

The datasets presented in the study are included in the article/supplementary material, further inquiries can be directed to the corresponding author/s.
